# Dexmedetomidine postconditioning provides renal protection in patients undergoing laparoscopic partial nephrectomy: A randomized controlled trial

**DOI:** 10.3389/fphar.2022.988254

**Published:** 2022-10-04

**Authors:** Lingling Jiang, Tao Zhang, Yang Zhang, Dexin Yu, Ye Zhang

**Affiliations:** ^1^ Department of Anaesthesiology and Perioperative Medicine, The Key Laboratory of Anesthesiology and Perioperative Medicine of Anhui Higher Education Institutes, The Second Hospital of Anhui Medical University, Hefei, China; ^2^ Department of Urology, The Second Hospital of Anhui Medical University, Hefei, China

**Keywords:** dexmedetomidine, ischemia postconditioning, laparoscopic partial nephrectomy, renal ischemia/reperfusion injury, renoprotection

## Abstract

**Background:** For localized disease, partial nephrectomy of small tumors continues to be the gold-standard treatment. However, temporary clamping is routinely performed during this process to control renal blood flow, which can cause renal ischemic/reperfusion injury. We evaluated whether dexmedetomidine postconditioning (DPOC) can reduce renal ischemic/reperfusion injury for patients receiving laparoscopic partial nephrectomy (LPN).

**Methods:** This randomized double-blind controlled trial included 77 patients who were scheduled for LPN at our hospital. Patients were randomly allocated to the DPOC or control group. DPOC was performed via intravenous administration of dexmedetomidine at 0.6 μg kg^−1^ for 10 min immediately after unclamping the renal artery. In the control group, saline was administered in place of dexmedetomidine under the same protocol. All participants underwent a 6-month follow-up. The primary outcome were the values of ^99m^Tc-DTPA-GFR in the affected kidney at one and 6 months post-LPN.

**Result:** The GFR values in the DPOC group (35.65 ± 4.89 ml min^−1^.1.73 m^−2^) were significantly higher than those the control group (33.10 ± 5.41 ml min^−1^.1.73 m^−2^; *p* = 0.022) at 1 month after LPN. There was no statistically significant difference in GFR value between the two groups at 6 months after LPN.

**Conclusion:** DPOC provides therapeutic benefits to LPN patients, at least on a short-term basis, by alleviating renal ischemic/reperfusion injury.

**Clinical Trial Registration**: Chinese Clinical Trial Registry, identifier [ChiCTR-TRC-14004766].

## Introduction

Laparoscopic partial nephrectomy (LPN) has become the standard treatment for small renal tumors (Huang et al., 2006). However, LPN typically requires temporary clamping of renal blood flow, which can prolong the warm ischemia time (Lane et al., 2008; Thompson et al., 2010). This procedure can also induce renal ischemic/reperfusion injury (IRI) and result in acute kidney injury (AKI). The kidney is highly sensitive to IRI due to its high energy demands and intricate microvascular network. Gill et al. (2007) reported that postoperative severe acute kidney injury, as defined by a glomerular filtration rate (GFR) < 15ml^−1^ min^−1^ or postoperative dialysis for 30 days, occurs in 3.6% of LPN patients. Lane et al. (2008) further indicate that a warm ischemia time of 20 min during LPN leads to substantially impaired renal function. It is therefore imperative to develop methods that reduce kidney injury in patients undergoing LPN surgery.

Ischemia preconditioning (IPRC) and ischemia postconditioning (IPOC) are two classic protective strategies commonly used in the treatment of IRI (Zhao et al., 2003; Xue et al., 2017). However, because the timing and onset of ischemia are generally unpredictable, it can be difficult to determine whether or not IPRC should be performed. IPOC, defined as rapid intermittent interruptions of blood flow in the early phase of reperfusion, has been proven to effectively protect kidneys from IRI in animal studies (Liu et al., 2007; Jiang et al., 2010; Tan et al., 2015). Although IPOC would seem to be the appropriate treatment, the temporary clamping of the renal artery in humans remains ethically controversial.

An alternative approach, known as pharmacological postconditioning (PPOC), mimics the protective effect of IPOC but uses pharmacological agents rather than clamping. Because alpha-2 adrenoceptors are widely found in the renal proximal and distal tubules, along with the peri-tubular vasculature, the alpha-2 adrenoceptor agonist dexmedetomidine is a logical candidate for use in IPOC. In addition to selectively activating alpha-2 adrenergic agonists, dexmedetomidine is known to have sedative, anxiolytic, hemodynamic stabilizing, and diuretic effects (Kim et al., 2019). PPOC using dexmedetomidine has been found to significantly reduce renal tubule damage and renal IRI in rat kidneys (Kocoglu et al., 2009; Gu et al., 2011; Balci et al., 2017; Kim et al., 2018). By inhibiting ischemia-induced excess noradrenalin secretion, dexmedetomidine may prevent the potential destructive effects of free-oxygen radical production (Kiliç et al., 2012). However, these promising results are based on *in vitro* and animal studies; it has not yet been determined whether dexmedetomidine postconditioning (DPOC) during LPN in human patients will reduce renal IRI. The goal of this study is accordingly to assess the effectiveness of DPOC in alleviating IRI in patients receiving LPN.

## Materials and methods

### Patients

This study was approved by the Ethics Committee of the Second Hospital of Anhui Medical University (LW201405). All protocols were registered at www.chictr.org.cn (Reference No: ChiCTR-TRC-14004766, 06/06/2014) and implemented in accordance with the principles of the Helsinki Declarations. The clinical trial was performed in compliance with CONSORT guidelines. All patients enrolled in the trial gave written informed consent. Patients enlisted in this randomized controlled trial were those diagnosed with kidney tumors and waiting for LPN surgery at our hospital between June 2014 and December 2015. The inclusion criteria for this trial were as follows: American Society of Anesthesiologists’ physical status of either 1 or 2, a maximum tumor diameter of 4–6 cm, and normal contralateral renal function (based on a differential renal function measurement of >40% obtained via radionuclide scintigraphy). We excluded patients older than 75 years and those with a history of one or more of the following diseases: heart, pulmonary, or hepatic disease, diabetes mellitus, or renal transplantation. We also excluded patients with acute renal disease, serum creatinine >1.5 mg dL^−1^, BUN >20 mg dL^−1^, or significant inflammation (chest radiographs suggest large-scale high-density shadows, white blood cells >1.0 × 10^9^ and patient temperature >38.5°C).

### Study design and randomization

Before surgery, the enrolled patients were assigned to either the DPOC group or control group in equal proportions. The randomization was performed by a computer program and the group assignments were sealed in sequentially ordered envelopes to obscure the random allocations. Nurses with knowledge of the group to which each patient was allocated prepared either dexmedetomidine or saline placebo in 50 ml syringes for the anesthesiologists, who administered the drugs and were blinded throughout the study. Accordingly, patients, surgeons, investigators and statisticians were all blinded to group allocation until the trial was complete.

In the DPOC group, a bolus dose of dexmedetomidine (0.6 μg kg^−1^) was infused for 10 min immediately after the renal artery was unclamped. The time point and length of administration, as well as the dose of dexmedetomidine, were selected according to previous studies (Tufanogullari et al., 2008; Kocoglu et al., 2009; Dahmani et al., 2010; Li et al., 2016; Balci et al., 2017; Cheng et al., 2018; Kim et al., 2018; Bunte et al., 2020), In the control group, saline was given in the same dose and according to the same protocol as the DPOC group.

### Anesthesia and surgery procedure

All surgeries were completed by one surgical team (D.X.Y. and T.Z.) according to established techniques described in the literature (Haber and Gill, 2006). After the patient arrived at the operating room, routine monitoring was established, which included electrocardiogram, pulse oximetry, end-tidal carbon dioxide (PetCO_2_), and invasive arterial pressure. General anesthesia was induced in patients using a target-controlled infusion of propofol (AstraZeneca S.P.A., Macclesfield, United Kingdom), aiming for a plasma concentration of 3.0–3.5 μg mL^−1^. The following additional routine anesthetics and drugs were also given: midazolam at 0.03 mg kg^−1^ (Yichang Humanwell Pharmaceutical Co., Ltd., Yichang, China), sufentanil at 0.5 μg kg^−1^ (Yichang Humanwell Pharmaceutical Co., Ltd., Yichang, China), and rocuronium at 0.6 mg kg^−1^ (N.V. Organon, Oss, the Netherlands). Following tracheal intubation, patients were ventilated with an oxygen and air mixture (FiO_2_ = 0.4) and a PetCO_2_ of 35–45 mmHg. Anesthesia was maintained with intravenous remifentanil (0.2–0.3 μg kg^−1^ h^−1^) and target-controlled infusions of propofol and cisatracurium besylate (0.1 mg kg^−1^ min^−1^). During the operation, bispectral index values in all groups were maintained at 45 ± 5 by regulating the propofol infusion rate. Following closure of the peritoneum, cisatracurium besylate infusion was stopped. Propofol and remifentanil were discontinued in inpatients after wound closure. Lactated Ringer’s solution was used in both groups to maintain a stable fluid balance. When the heart rate (HR) was less than 50 beats/min, patients were treated with 0.5 mg IV atropine. When the HR was higher than 120 beats/min, patients were treated with 20 mg IV esmolol. When the mean arterial pressure (MAP) was lower than 60 mmHg, phenylephrine 40 μg was intravenously injected; when the MAP was higher than 90 mmHg, nicardipine 0.25 mg mg was intravenously delivered. Following completion of the surgery, the patients were sent to the post-anesthesia care unit for recovery. A patient-controlled intravenous analgesia device was used to alleviate postoperative pain for all patients. Sufentanil (0.04–0.06 μg kg^−1^ h^−1^) was continuously administered for 48 h. For patients who still felt pain, a bolus dose of 0.15 μg Sufenanil was self-administered as needed (no more than once every 15 min).

### Collected data

Urological surgeons evaluated the tumor complexity with reference to the Preoperative Aspects and Dimensions Used for an Anatomical classification (PADUA) scoring system (Ficarra et al., 2009). To evaluate acute renal impairment, serum concentrations of neutrophil gelatinase-associated lipocalin (NGAL) and urinary concentrations of retinol-binding protein (RBP) were collected. Samples for the NGAL assay were collected intravenously at two and 6 h after surgery. These samples were then quantitatively monitored shortly after collection using the Triage NGAL assay (Alere, Inc., San Diego, CA). Urinary RBP was collected via catheterization before surgery, 24 h post-surgery, and 48 h post-surgery. The RBP was measured according to the micro-ELISA method using an automated instrument, TekTIME (BioMerieux, France). To evaluate long-term renal function, serum creatinine levels and the technetium ^99m^Tc-diethylene triamine pentacetic acid (DTPA) GFR were measured preoperatively, 1 month post-surgery, and 6 months post-surgery. An estimated GFR (eGFR) was calculated based on the updated CKD-EPI equation: eGFR (mLmin^−1^.1.73 m^−2^) = 175× (serum creatinine) ^−1.154^ × (age) ^−0.203^ × 0.742 (if female) × 1.212 (if black) (Levey et al., 2009). GFR values were estimated using the renal dynamic imaging method, with intravenous injection of ^99m^Tc-DTPA (purity >95%) produced by Jiang Yuan Pharmaceutical Factory (Jiangsu province, China), which was followed by measurement of the renal uptake ratio over time according to the Gates method. The GFRs were normalized by body surface area (1.73 m^2^). All surgeries were completed by one surgical team (D.X.Y. and T.Z.) in accordance with established techniques described in the literature, Perioperative adverse events were recorded.

The primary outcomes of this study were the values of ^99m^Tc-DTPA-GFR in the affected kidney at 1 and 6 months post-LPN. The secondary outcomes were as follows: serum NGAL concentration (measured preoperatively, 2 h post-surgery, and 6 h post-surgery); urinary RBP levels (measured preoperatively, 24 h after surgery, and 48 h after surgery); and serum creatinine levels and eGFRs (measured preoperatively, 1 month after LPN, and 6 months after LPN).

### Statistical analysis

Sample size calculations were based on a pilot study and historical data (Huang et al., 2013). To detect a clinically important difference between groups, 3.2 ml min^−1^.1.73 m^−2^ ^99m^Tc-DTPA GFR is sufficient, assuming an SD of 4.25 at 1 month after surgery in the affected kidney. The total sample size required is 76 when the level of significance (α) is 0.05 and the power is 0.9. Considering a dropout rate of 15% for various reasons, we estimated that 45 patients were needed in each group.

Variables were expressed as mean (SD), median with interquartile range (IQR), and percentages (%). Continuous variables were analyzed via ANOVA (for repeated measurements), student’s t-test, or Mann-Whitney U-test; moreover, categorical variables were analyzed using the Chi-square test (Prism9; GraphPad Software, San Diego, CA). *p* values <0.05 were considered statistically significant.

## Results

**FIGURE 1:**
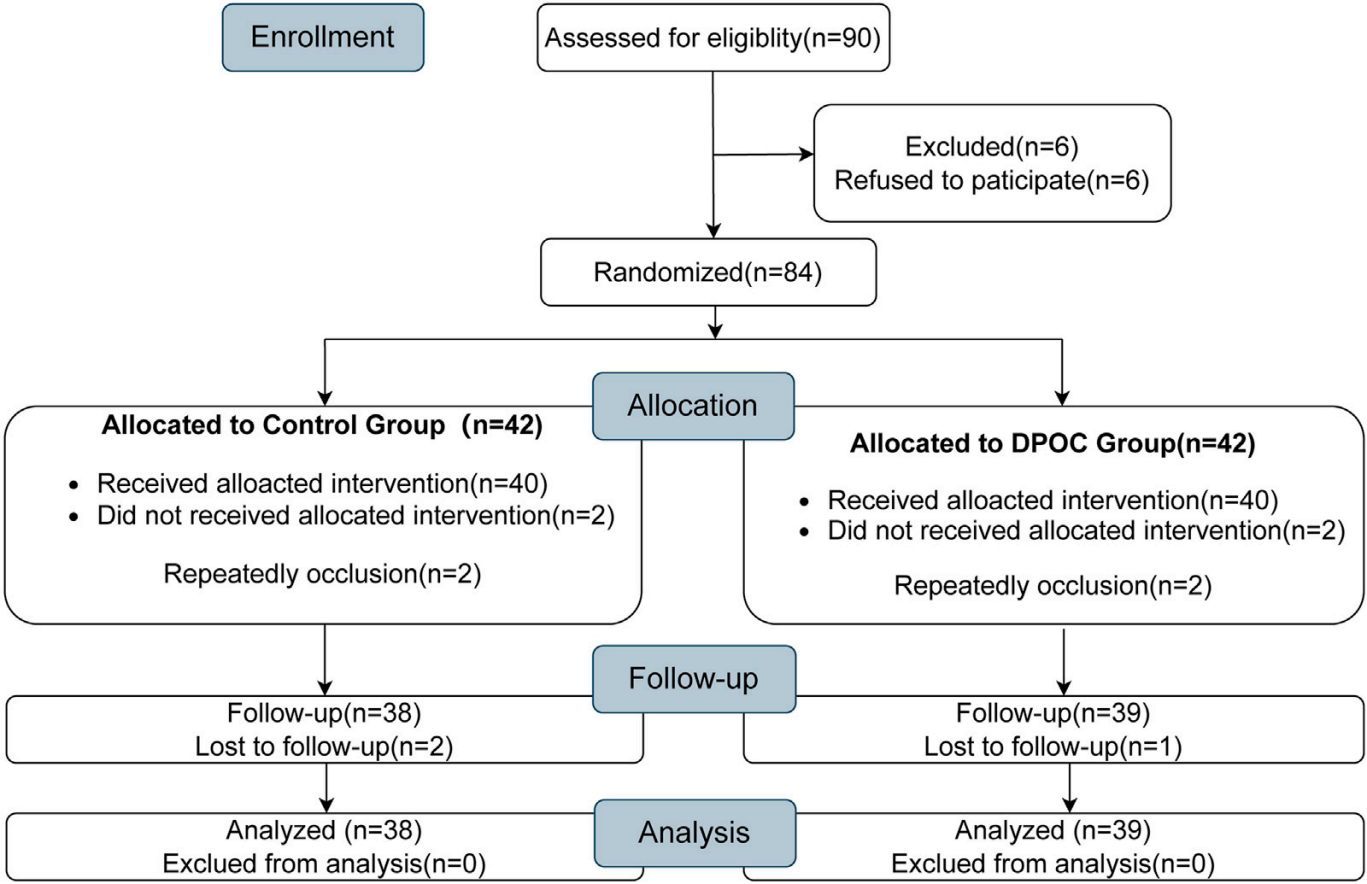
CONSORT diagram showing the flow of participants throughout each stage of the randomized trial.

A total of 90 patients were initially enrolled in this trial. Of these, six patients refused to participate, four patients experienced repeated occlusion during the surgery, and three patients were lost during the follow-up. After all patients listed above were excluded, a total of 77 patients completed this study: 38 in the control group and 39 in the DPOC group, as detailed in the CONSORT diagram in Figure 1. Adverse events included 4 cases of postoperative severe hematuria (2 patients in each group). 3 cases of postoperative peripheral hematoma (2 patients in the control group, 1 patient in the DPOC group). All the complications were cured by corresponding treatment.

The basic patient demographics and tumor characteristics of the two groups are listed in Table 1, while operative and postoperative characteristics are presented in Table 2. There were no differences in perioperative data between two groups (all *p* > 0.05).

**TABLE 1:** Baseline patient demographics and tumor characteristics.

Variables	Control(n = 38)	DPOC(n = 39)	*p*
Mean ± SD			
Age (years)	51.89 ± 10.90	52.18 ± 9.57	0.903
BMI (Kgm^−2)^	22.54 ± 2.43	22.79 ± 2.24	0.647
Preoperative GFR of affected kidney (ml^−1^min^-1^1.73m^−2^) (scintigraphy)	40.36 ± 5.28	39.66 ± 4.30	0.525
Preoperative GFR of contralateral kidney (ml^−1^min^-1^1.73m^−2^) (scintigraphy)	39.27 ± 6.26	38.65 ± 6.63	0.675
Tumor size (cm)	3.09 ± 0.82	3.26 ± 0.88	0.358
N (%)			
Male patients	26 (68.42)	28 (71.79)	0.807
Patients with hypertension	7 (18.42)	9 (23.08)	0.780
Right-side tumor	26 (68.42)	24 (61.54)	0.635
Pathological diagnosis			0.769
Renal Cell Carcinoma	32 (84.21)	31 (79.49)	
Benign	6 (15.79)	8 (20.51)	
Stage			0.780
T1a	30 (78.95)	32 (82.05)	
T1b	8 (21.05)	7 (17.95)	
PADUA score			0.981
6	6 (15.8)	5 (12.8)	
7	12 (31.6)	14 (35.9)	
8	13 (34.2)	12 (30.8)	
9	3 (7.9)	4 (10.3)	
10	4 (7.9)	4 (10.3)	
11	0 (0.0)	0 (0.0)	
12	0 (0.0)	0 (0.0)	

Note: Control = control group, DPOC = dexmedetomidine postconditioning group; BMI, body mass index; GFR, glomerular filtration rate; PADUA , preoperative aspects and dimensions used for anatomic urology.

**TABLE 2:** Operative and postoperative characteristics.

Variables	Control(n = 38)	DPOC(n = 39)	*P*
Mean ± SD			
Operative duration (min)	132.8 ± 16.69	130.8 ± 15.78	0.591
Anesthesia duration (min)	148.6 ± 16.86	146.4 ± 15.79	0.562
Operative fluid volume (ml)	1496 ± 279.2	1471 ± 233.4	0.664
Blood loss (ml)	362.4 ± 69.57	351.7 ± 64.92	0.497
Emergence time (min)	9.18 ± 2.09	9.33 ± 1.99	0.749
Median (IQR)			
Warm Ischemia Time	22 (16–26)	22 (18–26)	0.380
Pain score	3 (2–4)	3 (2–3)	0.073
Postoperative hospital stays	8 (7–9)	8 (8–9)	0.485
N (%)			
Agitation	3 (7.89)	2 (5.13)	0.622
PONV	2 (5.26)	1 (2.56)	0.541
Atropine	4 (10.53)	3 (7.69)	0.665
Esmolol	2 (5.26)	2 (5.13)	0.979
Phenylephrine	4 (10.53)	5 (12.82)	0.754
Nicardipine	3 (7.89)	4 (10.26)	0.719

Note: Control = control group, DPOC = dexmedetomidine postconditioning group; IQR, interquartile range.

**FIGURE 2:**
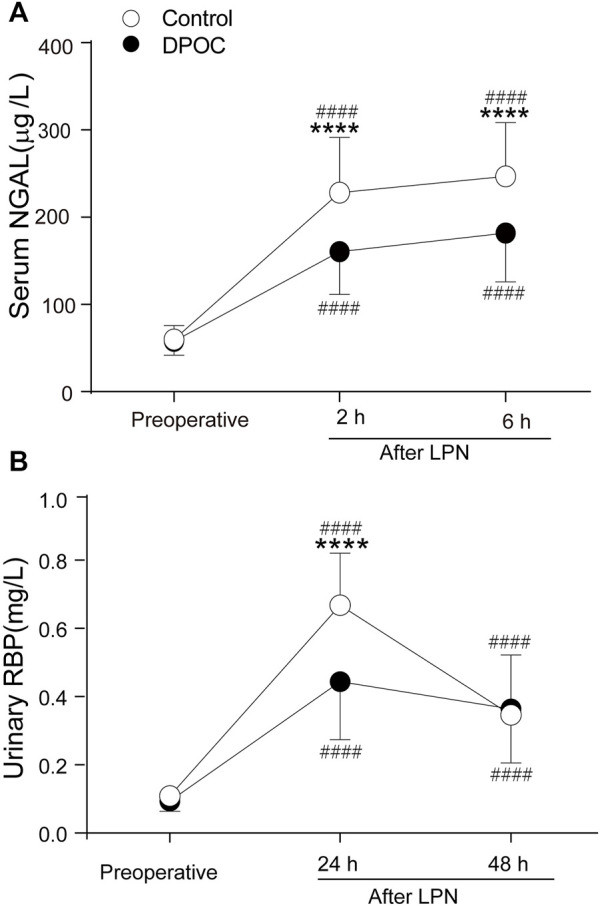
Levels of serum NGAL **(A)** and urinary RBP **(B)** before and after surgery between DPOC or control group. ****p < 0.0001 vs. control group. ####*p* < 0.0001 vs. Baseline. NGAL = neutrophil gelatinase-associated lipocalin; RBP = Urinary retinol-binding protein; Control = Control group; DPOC = dexmedetomidine postconditioning group.

There were no statistical differences between the control and DPOC groups in terms of preoperative serum NGAL concentrations. At two and 6 h after LPN, the level of serum NGAL [mean (SD)] in the control group was significantly higher than that in the DPOC group (control vs. DPOC: 228.2 ± 63.18 μg L^−1^ vs.160.4 ± 48.88 μg L^−1^ at 2 h after LPN; 247.0 ± 61.49 μg L^−1^ vs. 181.7 ± 55.96 μg L^−1^ at 6 h after LPN; *p* < 0.0001) (Figure 2A). LPN also triggered a notable rise in urinary RBP levels [mean (SD)] (from 0.11 ± 0.02 mg L^−1^ to 0.67 ± 0.15 mg L^−1^) at 24 h in the control group. However, this rise was less dramatic following DPOC treatment (from 0.10 ± 0.03 mg L^−1^ to 0.44 ± 0.17 mg L^−1^) at 24 h in the DPOC group; *p* < 0.0001 (Figure 2B). The levels of serum NGAL and urinary RBP were significantly higher after surgery in the two groups compared to the baseline (Figure 2).

**FIGURE 3:**
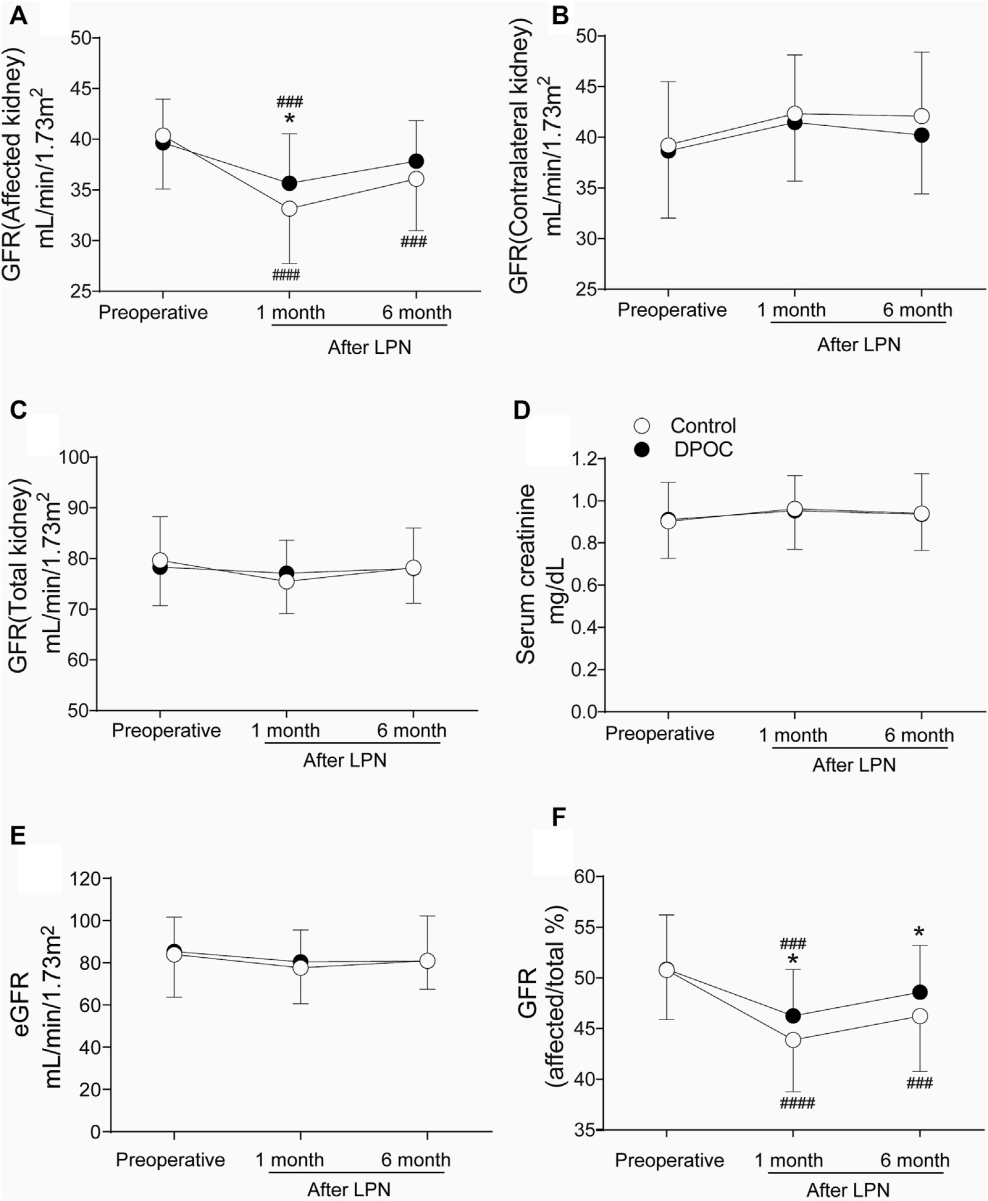
Renal function at one and 6 months after LPN between DPOC or control group. **(A)** GFR of the affected kidney; **(B)** GFR of the contralateral kidney; **(C)** GFR of the total kidney; **(D)** Serum creatinine; **(E)** Estimated glomerular filtration rate (eGFR); **(F)** Contribution change of the affected kidney to total renal function. All GFR values were evaluated by ^99m^Tc-DTPA renal scintigraphy. *p < 0.05 vs. control group. ###*p* < 0.001 vs. Baseline; ####*p* < 0.0001 vs. Baseline. GFR = Glomerular filtration rate.

Data regarding renal function (^99m^Tc-DTPA GFRs), serum creatinine levels, and eGFRs-are provided in Figure 3. The ^99m^Tc-DTPA GFR of the affected kidney in the DPOC group (35.65 ± 4.89 ml min^−1^ 1.73 m^−2^) was significantly higher than that in the control group (33.10 ± 5.41 ml min^−1^.1.73 m^−2^) at 1 month after LPN, *p* = 0.022 (Figure 3A). The trend shows that the ^99m^Tc-DTPA GFR of the affected kidney in the DPOC group was higher than that in the control group at 6 months after surgery, but there was no statistical difference between the two groups (*p* > 0.05). However, while the ^99m^Tc-DTPA GFR value in the affected kidney at 6 months after surgery (37.85 ± 4.01 ml min^−1^ 1.73 m^−2^) essentially returned to baseline (39.66 ± 4.30 ml min^−1^ 1.73 m^−2^) in the DPOC group (*p* > 0.05), the same value at the same timepoint (36.09 ± 5.10 ml min^−1^ 1.73 m^−2^) was still lower than the baseline (40.36 ± 5.28 ml min^−1^ 1.73 m^−2^) in the control group (*p* < 0.001). No significant differences were observed in the ^99m^Tc-DTPA GFR of the contralateral kidney and total kidney at one and 6 months after LPN (Figures 3B,C; *p* > 0.05). Furthermore, the serum creatinine levels and eGFRs in the control and DPOC groups did not significantly differ at various time points (Figures 3D,E; *p* > 0.05). To further confirm our findings, we calculated the GFR ratio for the affected kidney to total kidney (Figure 3F). This ratio was significantly higher in the DPOC group compared with the control group one and 6 months after LPN (46.27 ± 4.60% vs. 43.85 ± 5.11% at 1 month, *p* = 0.032; 48.59 ± 4.61% vs. 46.25 ± 5.47% at 6 months, *p* = 0.046). This ratio returned to baseline 6 months after LPN in the DPOC group compared to the value before operation (48.59 ± 4.61% vs. 50.87 ± 5.36% in the DPOC group, *p* > 0.05); however, the ratio in the control group at this timepoint was still significantly lower than pre-operation levels (46.25 ± 5.47% vs. 50.78 ± 4.89% in the control group, *p* < 0.001) (Figure 3F).

## Discussion

Since Zhao et al. (2003) first reported that IPOC can ameliorate myocardial ischemia/reperfusion, IPOC has been widely deployed to protect many organs from IRI (Jiang et al., 2010; Esposito et al., 2015; Ricca et al., 2015). Jiang et al. (2010) showed that IPOC can provide renal protection against IRI. Additionally, numerous studies focused on the IPOC mechanism have identified a range of endogenous and exogenous agonists that can produce similar therapeutic effects. For instance, dexmedetomidine can mimic IPOC’s renal protective effect by activating the PI3K-Akt survival cascade to induce cytoprotectant (Gu et al., 2011). Dexmedetomidine has been widely used in recent years as an anesthetic adjuvant during surgery due to its favorable perioperative hemodynamic stability and its intraoperative anesthesia-sparing effect (Ibrahim et al., 2019). Lempiainen, et al. (2014) suggested that dexmedetomidine preconditioning (DPRC), but not DPOC, restrain renal IRI. Nevertheless, several investigations have established the ability of DPOC to ameliorate heart, brain and kidney IRI (Kocoglu et al., 2009; Dahmani et al., 2010; Gu et al., 2011; Balci et al., 2017; Cheng et al., 2018; Bunte et al., 2020; Zhang et al., 2020). Our study is the first to conduct a randomized controlled trial on the renoprotection of DPOC in patients undergoing LPN. The data from this study suggest that DPOC can slow the decline of the GFR score and decrease the serum NGAL and urinary RBP values in the affected kidney. This indicates that DPOC can reduce acute renal impairment and improve at least short-term LPN outcomes.

Plasma NGAL provides a good diagnostic biomarker for AKI development within 48 h after surgery (area under ROC 0.78, 95%; CI 0.65–0.90) (Cruz et al., 2010). Our study shows that DPOC partially suppressed the increase in NGAL at 2 h and 6 h after LPN, while NGAL was substantially increased in the control group. Urinary RBP is considered a sensitive renal proximal tubular injury diagnosis index (Adiyanti and Loho, 2012; Obermüller et al., 2014). The normal urinary RBP content is very low (≤0.1 μg min^−1^), with a short plasma half-life of 12 h. Our results show that DPOC can slow the growth of urinary RBP postoperatively within 24 h. These findings suggest that DPOC conserves the function of the proximal tubules and thereby reduces AKI.

At 1 month and 6 months after LPN, no differences in total renal function—as reflected by serum creatinine, eGFR, and total GFR—were found between the two groups. This may be due to the compensatory GFR activity of the contralateral kidney, which proportionally masked the reduced GFR in the affected kidney. To resolve this issue, radionuclide imaging with ^99m^Tc-DTPA was used to assess whether DPOC can protect the affected kidney from injury. ^99m^Tc-DTPA GFR is widely used in clinical work due to its high accuracy and convenience. As a result, The ^99m^Tc-DTPA GFR in DPOC group is higher than control group at 1 month after surgery as 2.55 ml/min/1.73 m^2^ which is roughly equivalent to 7.6% of the whole ^99m^Tc-DTPA GFR. This dramatic improvement in ^99m^Tc-DTPA GFR for injury kidney may indicate a decrease in the risk for chronic kidney disease (CKD) and hemodialysis after discharge. However, no significant difference between the two groups was observed at 6 months after LPN. While gradual recovery of the kidney over the long-term may partially explain this result, there are also multiple factors that can influence long-term renal function, such as intraoperative blood loss (Simmons et al., 2012; Li and Liu, 2021). Nevertheless, DPOC caused a non-significant renoprotection trend, and the GFR value essentially returned to baseline at 6 months post-surgery. Furthermore, when assessing the contribution of the affected kidney to total renal function, the results showed that the contribution in the DPOC group was significantly higher than that in the control group. Overall, these findings indicate obvious renoprotection provided by DPOC, at least as assessed by short-term observations; a large-scale trial is required to evaluate the long-term effect.

While the overall mechanism by which DPOC conserves renal function is complex and unclear, the PI3K-Akt signaling pathway seems to play a critical role in DPOC against IRI (Dahmani et al., 2010; Gu et al., 2011; Cheng et al., 2018; Kim et al., 2018). PI3K-Akt is one of the survival cascades activated by alpha-2 adrenoceptors, which can decrease the expression of pro-apoptotic factors such as caspase-3 and Bax while up-regulating the expression of anti-apoptotic Bcl-2. Anti-inflammatory action is another possible mechanism by which DPOC conserves renal function during IRI. Gu et al. (2011) found that post-reperfusion treatment with dexmedetomidine can reduced plasma high-mobility group protein B1 (HMGB-1) levels after kidney ischemia/reperfusion. HMGB-1, a damage-associated molecular pattern released by dying cells during tissue ischemia, binds to toll-like receptor 4 (TLR-4), initiating a downstream NF-κB signaling cascade that promotes the synthesis of pro-inflammatory cytokine (IL-6, IL-1β, and TNF-α). Kim et al. (2018) demonstrated that dexmedetomidine reduced the elevation of the inflammasome components NLRP3, caspase-1 and IL-1β in a diabetic renal IRI model. In summary, the PI3K-Akt signaling pathway and anti-inflammatory effect may play key roles in DPOC, although the precise mechanism of DPOC needs to be further investigated.

The present study has a number of limitations. First, large-scale, multicenter clinical trials are required in order to further evaluate whether DPOC has long-term effects on renoprotection. Second, dexmedetomidine was administered as a single bolus dose over 10 min; larger doses of dexmedetomidine and longer infusion times should be tested in future to determine whether they can further improve renal function. Expanding follow-up studies to include more indicators (such as albuminuria or microalbuminuria) and additional timepoints (such as 2 to 3 months after LPN) would facilitate the construction of a more precise timeline of DPOC renoprotection. Finally, other isotopes such as ^99m^Tc-mercaptoacetyltriglycine-3 may enhance the accuracy in quantification of the GFR (Shirasaki et al., 2005).

## Conclusion

The findings of this study provide support for the theory that DPOC can alleviate AKI and provide short-term therapeutic benefits in reducing renal injury caused by LPN.

## Data Availability

The raw data supporting the conclusions of this article will be made available by the authors, without undue reservation.
